# Cardiovascular Cascade Genetic Testing: Exploring the Role of Direct Contact and Technology

**DOI:** 10.3389/fcvm.2016.00011

**Published:** 2016-04-19

**Authors:** Amy C. Sturm

**Affiliations:** ^1^Department of Internal Medicine, Division of Human Genetics, Ohio State University Wexner Medical Center, Columbus, OH, USA; ^2^Ohio State University Wexner Medical Center, Dorothy M. Davis Heart and Lung Research Institute, Columbus, OH, USA

**Keywords:** direct contact, cardiovascular genetics, familial hypercholesterolemia, genetic counselor, genetic counseling, cascade screening, cascade testing, genetic testing

Cascade screening is one of the more forceful demonstrations that molecular biology and genetics are not just a tool for researchers, but represent an important and by now essential component of good medical care.

– Peter J. Schwartz (1)

## Introduction

There is much attention and excitement in the current health care environment on the potential of precision medicine based on a patient’s genomic data. Today, what arguably remains as one of the most valuable and informative genetic tests is that of predictive testing for a known familial pathogenic variant. Predictive genetic testing determines whether the pathogenic variant previously identified in an affected family member(s) is present or not in relatives at risk. Previous research has documented that affected individuals undergoing genetic testing cite obtaining genetic information for others as being the most important, if not the only, motivation for undergoing genetic testing (2). Predictive, cascade testing is able to separate at-risk relatives who require vigilant serial screening from those who do not. For those with the predisposition, clinical screening allows for early identification of the family’s phenotype, which when present, may require lifelong medical therapy, implantation of devices, and/or other types of medical management. Relatives who test negative for the familial variant can typically be released from lifelong screening. In addition, it is also then known that their children are not at increased risk for the family’s disease. This approach can save the health care system, and the family itself, thousands of dollars. Cascade screening is imperative with “high-stakes” cardiovascular conditions, such as familial hypercholesterolemia (FH), long QT syndrome (LQTS), and other inherited arrhythmias, as well as other heritable cardiovascular phenotypes, including cardiomyopathies and aneurysms, where there is an increased risk for sudden cardiac death and severe morbidities such as heart failure.

The value of cascade screening for highly penetrant cardiovascular (and cancer) phenotypes has been acknowledged by public health officials. The United States Centers for Disease Control Office of Public Health Genomics classifies cascade screening of at-risk relatives for certain conditions, FH being one, as a Tier 1 genomic application, meaning it meets the criteria for analytic and clinical validity and utility and therefore has evidence supporting its implementation into practice (3). Cascade screening may include targeted genetic testing as well as clinical screening (e.g., lipid panel) of at-risk relatives.

This opinion provides a brief summary of research in this area and poses questions to facilitate future discussion regarding the potential for direct contact of at-risk relatives. As a practicing genetic counselor in clinical genetic medicine for 14 years who has provided genetic counseling and testing to thousands of families with heritable cardiovascular and cancer conditions, it is my opinion that more could be done to provide assistance to probands for at-risk relative notification and that genetic counselors are in the ideal position to facilitate cascade testing and lead forward-thinking research in this area (4).

## Cascade Screening: Where are We Now?

Cascade screening is a mechanism for identifying people at risk for a genetic condition by a process of systematic family tracing. It should begin with first-degree relatives (parents, siblings, and children) and then extend to second- and third-degree relatives in a stepwise, cascade fashion, moving through the pedigree in sequential steps as additional family members are diagnosed until all at-risk relatives have been screened (5). Cascade screening for FH is a cost-effective method for identifying new cases of FH (6–8). Cascade screening in families with inherited arrhythmia syndromes has been shown to lead to immediate prophylactic treatment, including drug treatment or implantation of pacemakers or cardioverter defibrillators (9). However, cascade screening is not effective unless at-risk relatives are first notified of their risk, the health implications of the inherited condition in their family, the availability of testing, with subsequent uptake. However, uptake of genetic counseling and predictive genetic testing has been shown to be inadequate (10). While there is support from payers, public health, and health care providers (HCPs) regarding the importance of cascade testing, how best to inform relatives of their risk and systematically implement cascade testing has yet to be determined.

Psychological, educational, geographical, and other barriers exist to family communication of genetic risk information. Ethical factors and family dynamics, including maintenance of confidentiality and privacy, potential for psychological harm and genetic discrimination (i.e., life insurance), balancing the right “not to know” with “duty to warn,” among others, must be considered (11). The currently recommended approach for FH, made by the International FH Foundation, includes the following: (1) the proband’s HCP should construct a pedigree that facilitates identification of at-risk relatives who should be offered testing; (2) the HCP should discuss risk notification with the proband; and (3) the proband should be provided with written information that includes general information about the family’s condition, the benefit, and availability of preventive therapies, emphasizes health consequences without testing, and be encouraged to share this with relatives (12). This approach should be taken with other highly penetrant autosomal dominant conditions. In one study specific to inherited arrhythmias and cardiomyopathies, probands were asked to distribute “family letters” containing information on risks, genetic and other screening tests, and preventive options to relatives at risk. In this study, 57% of informed relatives underwent screening (80% in arrhythmia families; 45% in cardiomyopathy families), and this was statistically significant when compared to the group where no family letter was provided (35%). While such “family letters” increased the number of relatives who presented for evaluation, over 50% in cardiomyopathy families and 43% overall of at-risk relatives had no documentation that they underwent cascade evaluation (13).

It has been suggested that it is not outrageous to expect that clinicians, once they have diagnosed a patient with a genetic arrhythmia, “track down” all at-risk family members and determine their genetic status (1). However, realistically, implementation of this approach is problematic since many health care systems do not support this type of family-centric care model. Specifically, a recent review presents health policy-related limitations faced in the United States to effective implementation of cascade screening and includes (1) a low rate of reimbursement for comprehensive genetic counseling services; (2) an individual, versus family-centric, approach to prevention and insurance coverage; (3) insufficient genetic risk assessment and knowledge by a majority of HCPs without genetics credentials; and (4) a shortage of genetics specialists (in rural areas especially) (14). In order to begin addressing and overcoming these challenges, research should be conducted demonstrating effectiveness of novel methods and tools that have the capacity to efficiently notify relatives of risk. These tools should provide education, offer support, and provide attainable next steps with calls to action so that probands can be assisted, and their relatives can understand their own risk and be supported to act on it.

## Direct Contact in Cascade Screening: Should We Take a More Active Approach?

Different methods of informing relatives of risk exist including (1) proband, or family-mediated, contact; (2) proband, or family-mediated, contact *with assistance* (provision of materials, such as a family letter or other written information aids, by the HCP to the proband); and (3) direct contact of at-risk relatives by the clinical service itself.

Research suggests that clinical providers may take an active approach and directly contact relatives to notify them of their risk without compromising privacy or autonomy, with significantly higher numbers of relatives whose genetic status is clarified for greater efficiency, and with high levels of acceptability (15–18). A thematic analysis of FH proband interviews found that probands believed they had insufficient authority or control to persuade family members to attend screening and that they welcomed greater assistance from the clinic for contact with relatives (19). Also in support of direct contact is increased accuracy, as errors may occur in proband-mediated transmission of genetic testing result information through families (20). However, a prior study found that FH patients who expressed a preference regarding cascading method favored indirect contact because they considered it less threatening to family members (21). A genetic counseling intervention study that offered direct contact to the index patient as a last option for assistance in informing at-risk relatives reported no uptake; only eight index patients were offered this service, however, and none of the patients in this study had cardiovascular phenotypes (22). A recent literature review concluded that most studies support direct contact of relatives *via* letter mailed from the provider and that provider-initiated communication more often resulted in relatives being tested compared to other methods of communication (16).

Regarding additional Tier 1 conditions, a prospective study of families with *BRCA* mutations associated with Hereditary Breast Ovarian Cancer syndrome compared proband-mediated contact to a direct contact intervention protocol that included a letter and subsequent phone call to at-risk relatives (17). This study concluded that the direct contact protocol nearly doubled the number of relatives tested and was also found to be psychologically safe. A direct contact study in families with Lynch syndrome, or hereditary non-polyposis colorectal cancer, demonstrated high approval in those who consented to participate, with a third of newly diagnosed mutation carriers having cancer identified in their first post-test colonoscopy. This type of data demonstrates acceptability of direct contact risk notification programs, as well as efficacy, feasibility, and also ethical responsibility.

From the perspective of those potentially at risk, a study conducted in Australia assessing community members’ viewpoints showed that over 90% of respondents indicated their desire to be informed about a familial risk of FH and to be offered screening, with evidence of strong community support for direct contact by an FH clinic (23). The “right *to* know” must also be considered.

## Future Directions

Research evaluating genetic counseling interventions focused on strengthening family communication, the number of relatives informed of risk, and the impact on uptake of genetics services is ongoing and will help inform future efforts (22, 24). A randomized controlled trial studying whether a specifically designed genetic counseling intervention that included telephone support up to three times post new genetic diagnosis showed no overall significant difference for the level of family communication between the intervention and control groups (25). In this study, the level of family communication was the highest for conditions with appropriate treatments or active surveillance, such as LQTS and hypertrophic cardiomyopathy. While promising, the level still only reached ~30%. These data again beg the question regarding the potential role for direct contact, especially in “high stakes” conditions.

Most, if not all, of the research conducted to date specific to direct contact has been done outside the United States. Therefore, there is a real need for research to determine whether direct contact methods would be acceptable to probands, at-risk relatives, and HCPs within the United States. How many probands might indeed welcome and appreciate this assistance and support and opt in to programs that work *with* and/or *for* them to assist in disclosure of risk information to relatives? This opinion piece does not propose that we break probands’ confidentiality and throw privacy to the wind. Instead, it hopes to promote additional conversation and brainstorming that may lead to the development and testing of innovative models of care for probands with highly penetrant, yet manageable conditions. The ultimate goal is that we will have greater impact in our work with these families where there are clear risk-reducing interventions. Probands and family members should be engaged in shaping these models and the research testing them, starting now!

The next question becomes, what is feasible now in the landscape of our current health care system? Can we systematize the collection of informed consent from probands to directly share their protected health information with relatives for which they provide the clinic contact information? Can we offer probands active assistance in family communication of genetic risk information? In the pediatric setting, is there a role for standardized direct contact of HCPs caring for the at-risk children in our pedigrees with FH, other Tier One conditions, and beyond? This may be a service welcomed by the affected parent proband, who may appreciate greater assistance in coordination of care for their at-risk children and other pediatric members of their family.

Advances in web-based technologies and novel models for the delivery of genetic counseling may be able to bring cascade testing more effectively and efficiently to larger numbers of at-risk relatives. For example, home-based online genetic counseling sessions for cardiovascular genetic cascade screening can be effective (26), allowing at-risk individuals to access their genetic risk information at the time of their choosing and without having to travel to a hospital or clinic, a barrier mentioned previously. In addition, interactive e-learning and decisional support e-tools available *via* informative websites and mobile applications have been used in pre-test genetic counseling with high knowledge and satisfaction, leading toward the “e-informed” patient (27). Mobile health applications have been shown to result in more “activated” patients – defined as individuals who believe their roles are important, that they have the confidence and knowledge needed to take action, and that they can engage in health-promoting behaviors (28), such as predictive genetic testing. Probands with higher activation may lead toward more at-risk relatives notified of their risk. In turn, e-learning information, such as an informational video about the family’s inherited cardiovascular disease, could then be delivered to relatives, who may then become activated themselves to pursue cascade testing.

The power of preventive genetic and genomic information is real – that is not the question. How to ensure this information gets into the hands of all that need it, including children, however, needs more active attention.

In conclusion, a powerful quote from Newson and Humphries (11): “Our biology does not stop: the risk of developing coronary heart disease as a consequence of FH will still be present, even if relatives live in ignorance.”

## Author Contributions

The author confirms being the sole contributor of this work and approved it for publication.

## Conflict of Interest Statement

The author declares that the research was conducted in the absence of any commercial or financial relationships that could be construed as a potential conflict of interest.
